# Comparing self-reported ethnicity to genetic background measures in the context of the Multi-Ethnic Study of Atherosclerosis (MESA)

**DOI:** 10.1186/1471-2156-12-28

**Published:** 2011-03-04

**Authors:** Jasmin Divers, David T Redden, Kenneth M Rice, Laura K Vaughan, Miguel A Padilla, David B Allison, David A Bluemke, Hunter J Young, Donna K Arnett

**Affiliations:** 1 Department of Biostatistical Sciences, Wake Forest University School of Medicine Winston-Salem, North Carolina 27157, USA; 2 Department of Biostatistics, Section on Statistical Genetics, University of Alabama at Birmingham Birmingham, Alabama 35294, USA; 3 Department of Epidemiology University of Alabama at Birmingham Birmingham, Alabama 35294, USA; 4 Department of Biostatistics University of Washington Seattle, WA 98195, USA; 5 Division of Internal Medicine and Department of Epidemiology, University School of Medicine Baltimore, MD 21287, USA; 6 Department of Radiology and Medicine John Hopkins University School of Medicine Baltimore, MD 21287, USA

## Abstract

**Background:**

Questions remain regarding the utility of self-reported ethnicity (SRE) in genetic and epidemiologic research. It is not clear whether conditioning on SRE provides adequate protection from inflated type I error rates due to population stratification and admixture. We address this question using data obtained from the Multi-Ethnic Study of Atherosclerosis (MESA), which enrolled individuals from 4 self-reported ethnic groups. We compare the agreement between SRE and genetic based measures of ancestry (GBMA), and conduct simulation studies based on observed MESA data to evaluate the performance of each measure under various conditions.

**Results:**

Four clusters are identified using 96 ancestry informative markers. Three of these clusters are well delineated, but 30% of the self-reported Hispanic-Americans are misclassified. We also found that MESA SRE provides type I error rates that are consistent with the nominal levels. More extensive simulations revealed that this finding is likely due to the multi-ethnic nature of the MESA. Finally, we describe situations where SRE may perform as well as a GBMA in controlling the effect of population stratification and admixture in association tests.

**Conclusions:**

The performance of SRE as a control variable in genetic association tests is more nuanced than previously thought, and may have more value than it is currently credited with, especially when smaller replication studies are being considered in multi-ethnic samples.

## Background

The use of self-reported race and ethnicity (SRE) in genetic and epidemiologic studies has been much discussed in the literature [1-8]. Some researchers proposed to completely ban their utilization in these studies claiming that race and ethnicity are poorly defined social constructs with weak biologic and genetic basis [2,3,9]. Others, however, have argued that completely disregarding racial and ethnic differences in genetic and epidemiologic studies may not be appropriate, since these differences can be useful when generating and exploring new hypotheses regarding the effect of environmental and genetic risk factors and their interaction on important medical outcomes [1,9].

Some studies have found SRE to be closely related to an individual's genetically estimated ancestry proportions [8,10] and have suggested that SRE may provide adequate control against type I error inflation and/or loss of power due to population stratification and admixture in genetic association tests. However, it has also been shown [5,11] that while SRE may be sufficient to predict the continent or subcontinent on which an individual's ancestors were born, genetic markers may provide a finer genetic ancestry measure capable of capturing more subtle variation within ethnic groups. In fact, most investigators currently rely on a genetic measure of an individual's ancestral background as a control variable against confounding due to population stratification and admixture in genetic association tests instead of the SRE. This use of genetic background measures is particularly common in large endeavors such as in genome-wide association studies. However, in smaller candidate gene studies, investigators have asked whether accounting for SRE alone might be sufficient to control for the confounding effect, especially when the number of markers needed for controlling against confounding effects that are caused by population stratification and admixture is large compared to the number of variants to be tested.

We used ancestry informative markers (AIMs) and phenotypic data on left-ventricular hypertrophy (LVH) collected in the context of the Multi-Ethnic Study of Atherosclerosis (MESA) to address two related questions: (1) what agreement is there between SRE and clusters created based on the genotyped AIMs? (2) In multi-ethnic genetic association studies, does SRE provide comparable type I error control to that provided by genetic ancestry background measures, such as individual ancestry proportions or genetic background scores? To address these two questions we compared three sets of measures; SRE, individual ancestry proportion (IAP) estimates obtained using STRUCTURE [7,12], and a genetic background score (GBS) which we define below. We then tested for their genetic association with left ventricular mass and its related systolic functional counterpart, the left ventricular ejection fraction. These two phenotypes are known to vary considerably between ethnic groups. We also used these phenotypes as the basis to generate plasmodes [13,14] and to illustrate the potential for type I errors when genetic association studies are conducted on phenotypic variables that are differentially distributed among the 4 ethnic groups (African-Americans (AA), Chinese-Americans (CA), European-Americans (EA) and Hispanic-Americans (HA)) represented in MESA.

LVH is a condition where the ventricular mass increases as existing cells of the LV enlarge or hypertrophy [15]. LVH is one of the most potent risk factors for cardiovascular disease, particularly ischemic heart disease and heart failure [16,17], and its reversal has recently been shown to reduce the rate of cardiovascular events independently of the blood pressure level [18]. Risk factors associated with LVH include age, gender, hypertension, obesity, and diabetes [18,19]. There is significant evidence of ethnic differences in the distribution of LVH. The rate of occurrence of LVH in EA is approximately 16%, compared to 33-43% in AA [20-23]. Many of the risk factors for LVH also differ across ethnic groups and may partially account for the observed ethnic differences in LVH. However, given the number of potential determinants of LVH, there are plausibly several genes acting independently or synergistically to increase risk for LVH in different populations. A substantial amount of work has been done and published on LV mass and other LVH related phenotypes using data collected in MESA [24] and other studies.

## Results

### Agreement between self-reported ancestry and the genetic background scores

**Figure 1:**
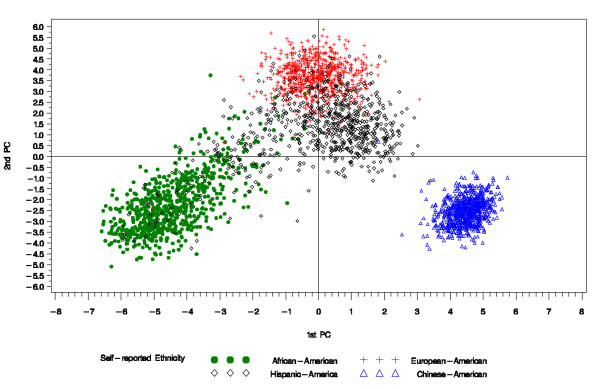
**Principal component analysis in 2D of the MESA AIMs**. The Hispanic-American group seems to be more heterogeneous than the remaining groups with fairly large number of self-identified Hispanics being assigned to the same cluster as the self-identified European-Americans.

Ward's minimum variance and the K-means clustering algorithm applied on the 96 AIMS yielded similar clusters. Here we present only the results from the K-means algorithm. Figure 1 shows 4 clusters: the CA cluster, represented by the triangles in the graph, is completely separated from the remaining clusters with very few cases where SRE and the clustering algorithm disagree; the AA cluster, represented by the circles, is also well delineated, as is the EA cluster represented by the plus signs. The HA cluster, represented by the diamonds, appear to be the most heterogeneous group with most of the self-identified Hispanics being clustered with the AA and the EA. This finding is not surprising since Hispanics in the MESA were recruited in the New York City and Los Angeles areas. The New York City area has a large population of Caribbean Hispanics who are known to have a significant degree of African ancestry, whereas Hispanics in the Los Angeles are mostly of Mexican descent with limited African ancestry [25]. Table 1 shows the agreement between self-reported ethnicity and the observed clusters. The agreement coefficient *κ *between the two classification procedures is about 0.83 when all four self-reported ethnicities are analyzed jointly. However, the agreement between these two methods is almost perfect (*κ *= 0.98) when the self-reported Hispanics were not considered in the analysis. The marginal homogeneity test, which in this case is testing the null hypothesis that 2 classification methods are consistently assigning the same individuals to the same cluster, is rejected at the 0.05 significance level when the self-reported Hispanics are included in the analysis $\left(\chi_{1}^{2}=9.8,pvalue=\text{0}\text{.001}\right)$. We failed to reject the same hypothesis at that same significance level when we repeated the analysis excluding the self-reported Hispanic-Americans $\left(\chi_{1}^{2}=3.6,pvalue=\text{0}\text{.06}\right)$. This secondary analysis reinforces the previous results observed with the kappa coefficient. In MESA, there is a very high agreement between self-reported ethnicity and individual ancestry estimates computed using genotyped AIMs for Americans who identify themselves as either of European, African or Chinese descent. SRE seems to be a less reliable indicator for Hispanic-Americans. We note that the term Hispanic-Americans, in general, refers more to social-cultural factors than to a genetically homogeneous group of people. These findings have motivated the genotyping of additional ancestry informative markers in the MESA study in an effort to better tease out genetic variations among the self-reported HA. A set of ancestry informative markers that allow for a better distinction between individuals coming from Latin American have since been added to the set of AIMs in order to refine the individual ancestry estimates computed in this sample.

**Table 1:** Agreement between self-reported ethnicity and the 4 observed clusters

Self-reported Ethnicity	Assigned Ethnic group				Total
		
	AA	CA	EA	HA	
African American	637	0	8	67	712

Chinese American	0	717	1	0	718

European American	1	0	630	81	712

Hispanic American	69	3	135	498	705

**Tota**l	707	720	774	646	2847

### Agreement between self-reported ancestry and the ancestry proportion estimates

**Figure 2:**
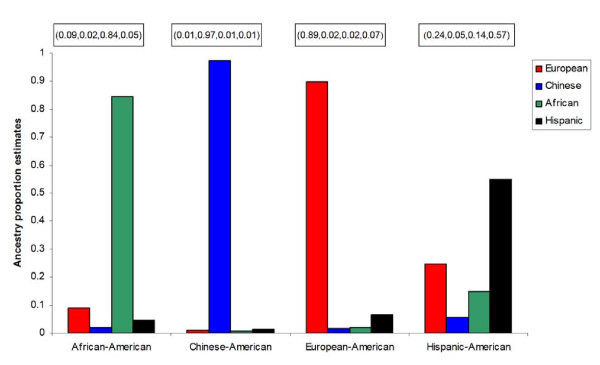
**Comparison between self-reported ancestry and structured estimated ancestry proportion**. Ideally without admixture, each group would be represented by just one bar. That is if the vector *Q *= (*q*_1_,*q*_2_,*q*_3_,*q*_4_) represents the ancestry score for each individual in the dataset, *Q *should have 3 of its components equal to 0 and one component equal to 1. As can be seen from this figure, this is not the case. Self-reported ancestry is again less reliable for the Hispanic-American population since its graph seems to be the farthest away from the ideal situation.

We also ran the program STRUCTURE to obtain individual ancestry proportion estimates assuming 4 ancestral populations. Let *Q *= (*q*_1_,*q*_2_,*q*_3_,*q*_4_) denote the ancestry proportion of an individual in the dataset where *q_i _*represents the proportion of alleles that this individual has inherited from the *i^th ^*ancestral population. *Q *therefore estimates, for each individual, the proportions of African, Chinese, European and American ancestry estimated based on the genotyped AIMs. In general, for individuals who have not experienced a recent admixture event in their lineage, it is expected that the *Q *vector will have 3 of its components very close to 0 and the fourth component close to 1. All significant deviations from these numbers can be seen as either evidence of recent admixture events, a sign of discrepancy between self-reported ancestry and estimated ancestry given that each MESA participant self-identified with only one ethnic group, or weak resolution due to limited ancestry informativeness content of the marker panel. The average ancestry proportion estimate is given for each self-reported ethnic group in Figure 2. This figure shows very little difference between self-reported ancestry and estimated ancestry proportion for AA, CA and EA. However, one can again observe more discrepancy between self-reported HA and their ancestry proportion estimates, which confirms the initial results observed with the genetic background scores. This result is not entirely surprising and is usually the first one cited to discourage reliance on SRE only as a control variable in genetic association tests. Although self-reported ethnicity can help group individuals coming from geographically distant regions, it does not always distinguish those who have mixed origins. However, as will be seen below, it seems that in multi-ethnic samples, misclassification errors in the SRE and the number of ancestral populations represented in the samples play a more important role on the performance of SRE as a control than the actual ancestry proportions. Different linkage disequilibrium may also affect the performance of SRE and GMBAs in general, but that is a separate issue.

### Distribution of body surface adjusted (BSA) LV mass and the LV ejection fraction

The summary statistics for the distribution of BSA LV mass and the LV ejection fraction by self-reported ethnicity is given in Table 2. The p-value of the Wald 3 degrees of freedom test for equality of the mean LV mass in all 4 groups is 7 × 10^-14^, which shows strong evidence that the mean LV mass is different between the 4 self reported ethnic groups. The association of SRE with the distribution of the LV ejection fraction is more pronounced compared to what is observed with adjusted LV mass. Although the observed mean and standard deviation of the LV ejection fraction in each ethnicity appear to be very close, its overall distribution varies in the 4 ethnic groups. The Kruskal-Wallis test comparing this distribution in the 4 ethnic groups has a p-value of 2 × 10^-24^, which strongly suggests that the distributions are different in the 4 self-reported ethnic groups. These two results confirm the presence of the previously identified ethnic specific differences in the distribution of both BSA adjusted LV mass and the LV ejection fraction. We also note that after standardizing both phenotypic variables, SRE explains only 3% of the variation explained in LV mass while it explains 19.5% of the variations in LV ejection fraction. Therefore, it is expected that variations in ethnicity will play a more important role in determining the level of LV ejection fraction compared to adjusted LV mass.

**Table 2:** Distribution of adjusted LVH and adjusted ejection fraction by self-reported ethnicity

Self-reported Ethnicity	BSA adjusted LV mass				LV ejection fraction			
	
	AA	CA	EA	HA	AA	CA	EA	HA
N	498	591	544	519	498	591	544	519

Mean	79.9	73.8	75.8	80.7	68.4	72.3	68.5	68.7

Standard deviation	17.0	13.6	15.5	17.6	7.6	6.1	7.4	7.5

Minimum	37.4	42.2	40.4	34.6	40.6	45.3	22.2	28.1

Q1	68.3	64.7	64.9	68.5	63.5	68.4	64.2	64.4

Median	77.9	72.1	73.8	78.0	69.0	72.9	68.6	69.9

Q3	89.7	81.5	85.6	89.6	73.6	76.3	73.6	73.9

Maximum	146.6	180.4	163.6	153.1	88.1	81.6	86.6	84.4

### Association between the AIMs and body surface adjusted LV mass and LV ejection fraction

It is known that population stratification can lead to confounding issues in genetic association studies [26]. To assess the magnitude of this confounding effect in the MESA study, we tested each SNP for association with LV mass and the LV ejection fraction accounting for SRE, IAP and GBS respectively. We observed better agreement between the p-values obtained with GBMAs than we did between either one of them with SRE. For example, the Spearman correlation coefficient between the p-values observed with the three pairs of control variables (SRE, IAP), (SRE, GBS) and (IAP, GBS) are respectively 0.85, 0.89 and 0.98. This result suggests that SRE is performing as well as the IAP and GBS although the p-values observed when controlling for the GBMAs are closer to each other, which is to be expected. The type I error rate observed with the resampling procedure are shown in Table 3 for LV mass and Table 4 for the LV ejection fraction. These tables show that SRE, at least in the context of the MESA, offers the same level of protection against type I error due to population and/or admixture than the GBMAs. Moreover, the association tests, for which SRE was used as a control variable, seemed to be a bit conservative. It is worth noting that when confounding due to population stratification is ignored, the type I error inflation that ensued is respectively 6 times the nominal rate for BSA adjusted LV mass and 14 times this rate for the LV ejection fraction (result not shown).

**Table 3:** Type I error associated with the test for association between LV mass and the 96 AIMs

Control variable	Average Type I error	Standard error	Observed minimum Type I error	Observed Maximum Type I error
Individual admixture estimates	0.033	0.006	0.0105	0.042

Principal components	0.048	0.0074	0.021	0.052

Self-reported Ethnicity	0.037	0.006	0.021	0.052

Ignoring confounding	0.320	0.022	0.221	0.357

**Table 4:** Type I error associated with the test for association between LV ejection fraction and the 96 AIMs

Control variable	Average Type I error	Standard error	Observed minimum Type I error	Observed maximum Type I error
Individual admixture estimates	0.038	0.0058	0.021	0.042

Principal components	0.048	0.007	0.021	0.052

Self-reported Ethnicity	0.042	0.004	0.031	0.053

Ignoring confounding	0.705	0.009	0.694	0.737

Note that when the confounding effect is ignored the type I error is about 14 times the nominal rate. However, controlling for Self-reported ethnicity alone in this case is sufficient to keep the type I error rate at its nominal value.

As mentioned above, the second set of simulation studies was designed to better understand when SRE might perform well as a control variable in genetic association tests.

**Figure 3:**
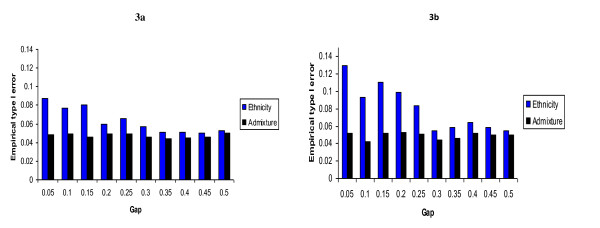
**Empirical type I error observed when self-reported ethnicity (assuming no misclassification error) and individual ancestry proportion are respectively used as control variables in the association test**. Figure 3a shows the effect of the gap in the distribution of individual admixture on type I error rate when controlling for admixture and ethnicity when the effect size is equal to 0.5.  One can observe on figure 3a that there is a slight type I error inflation even when true ethnicity is used instead of the true ancestry proportion. This inflation decreases as the gap in the admixture distribution increases. The admixture distribution in this case is univariate as would be observed for an admixed population derived from intermating between exactly 2 ancestral populations. Figure 3b shows higher type I error inflation rate when the effect size is equal to 1.

These simulations showed that when the confounder is univariate - that is, when there are exactly 2 ancestral populations, some type I error inflation may still occur when SRE is used as a control variable. We observed this inflation even in the absence of misclassification error. If there is a discontinuity point, as would most likely be the case when a sample of African Americans and European Americans is collected, the bigger the gap in the observed ancestry proportion the smaller the type I error inflation. Therefore, it is safe to conclude that the inflation rate depends on the composition of the sample. For example, when the study sample is comprised of admixed individuals derived from intermating between exactly 2 ancestral populations, the type I error inflation can be very small when the study sample is composed of individuals whose ancestry proportions are near the extreme values (near 0 or 1). This type I error inflation becomes substantial when the sample is composed of individuals whose ancestry proportion is near 50%. Figure 3 shows the observed type I error for different gap values assuming that the marker has an effect size of 0.5 (Figure 3a) and l standard deviation (Figure 3b). Self-reported ethnicity does not always correspond to true ethnicity. Some misclassifications or discrepancies between self-reported ethnicity and true ethnicity are likely to occur. The amount of type I error inflation that occurs when ethnicity is reported with error is an increasing function of the misclassification rate. The effect of various misclassification rates is displayed in Table 5 for gap values varying from 0.05 to 0.5 by 0.15 and misclassification rate varying from 0.05 to 0.15 by 0.025. Three observations can be made from this table: (1) as noted above, controlling for true ethnicity even if it were known can still lead to type I error inflation depending on the distribution of individual admixture in the sample, (2) the higher the misclassification rate, the higher the type I error inflation rate, and (3) the effect of misclassification error on type I error inflation is not uniform; the bigger the gap in the observed distribution of admixture, the more negative its effect will be on the type I error inflation.

**Table 5:** Observed type I error rates when controlling for individual ancestry estimate, true ethnicity and self-reported ethnicity assuming various misclassification error rates when admixed population results from intermating between exactly two ancestral populations

Gap	Misclassification rate	Ethnicity	Admixture	SRE
	0.05	0.067	0.046	0.402
	
0.05	0.075	0.087	0.041	0.46
	
	0.1	0.089	0.061	0.432
	
	0.125	0.083	0.059	0.42
	
	0.15	0.071	0.047	0.427

	0.05	0.061	0.054	0.517
	
0.2	0.075	0.075	0.059	0.52
	
	0.1	0.077	0.051	0.518
	
	0.125	0.063	0.045	0.517
	
	0.15	0.067	0.055	0.492

	0.05	0.058	0.05	0.631
	
0.35	0.075	0.06	0.056	0.622
	
	0.1	0.067	0.051	0.657
	
	0.125	0.06	0.052	0.62
	
	0.15	0.054	0.044	0.642

	0.05	0.064	0.066	0.752
	
0.5	0.075	0.061	0.063	0.767
	
	0.1	0.043	0.038	0.787
	
	0.125	0.05	0.043	0.783
	
	0.15	0.062	0.064	0.765

**Figure 4:**
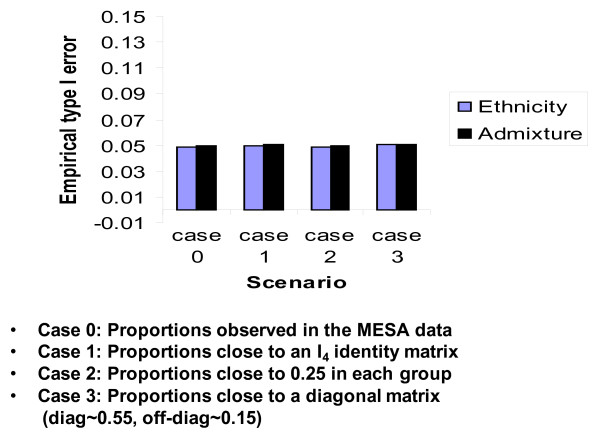
**Observed type I error rate when the sample is multi-ethnic**. Controlling for self-reported ethnicity leads to the preset significance level. It seems the value of ancestry proportions matters less.

The type I error rates observed for simulation 3 are displayed in Figure 4. SRE performed as well as the GBMAs in the multi-ethnic sample. Contrary to the univariate case, where the distribution of the ancestry proportions represented in the sample appeared to affect the performance of SRE, these proportions seem to matter less in a multi-ethnic sample. In fact, SRE performed as well in all 4 cases described in simulation 2 although the ancestry proportions are quite different in each scenario. However, misclassification errors remain a significant determinant of the level of type I error control that is achieved.

**Figure 5:**
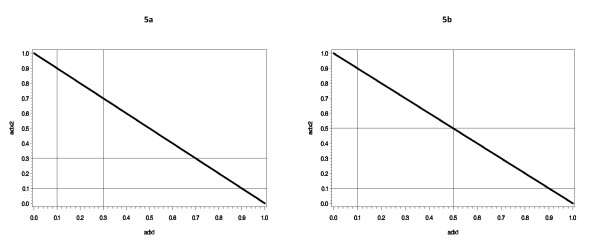
**Boundaries for simulated ancestry proportions when there are 3 ancestral populations**. Figure 5a shows 4 valid regions, and if one decides to assign ethnicity according to the maximum value of the vector (*adx*_**1**_, *adx*_**2**_, *adx*_**3**_), it is not exactly clear what the correct ethnicity assignment should be for the individuals whose ancestry proportions fall in region IV. There is no such ambiguity in figure 5b.

**Figure 6:**
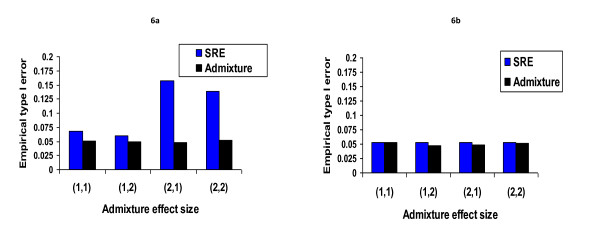
**Observed type I error rate when there is misclassification error between SRE and admixture**. We observed significant type I error inflation in figure 6a that is due to the possibility for misclassification error described in figure 5a. Once we the ambiguity is removed (figure 5b) and there is no possible misclassification errors, we see that observed type error rate is maintained at the preset significance level. The distribution of allele frequencies in the 3 ancestral populations appears to also affect the degree of confounding that occurs, such that the type I error inflation due to misclassification errors is worst for some cases than it is for others. For example, when we changed the allele frequency of marker G_2_ from (0.2, 0.4, 0.6) to (0.4, 0.2, 0.6) to make it independent but identically distributed with G_1_ then the observed type I error rate dropped from 0.165 to 0.07. We repeated the same experiment by keeping the frequency of G_1_ fixed and changing the frequency of G_2_ to (0.1, 0.3, 0.5). The observed type I error is now 0.18, and again by changing the order of the first 2 components of the allele frequency vector, this type I error is reduced to 0.065. The type I error associated with the scenario described in figure 5b remain around the preset threshold of 0.05 independently of the choice of allele frequencies in the 3 ancestral populations.

We ran additional simulations studies in order to better understand the effect of misclassification errors on the type I error rate. We considered two scenarios that are described in Figure 5. Figure 6 displays the type I error rate observed when the admixed sample has 3 ancestral populations. Figure 6a shows that when there is misclassification, as would be the case under the scenario illustrated in Figure 5a, SRE would not perform as well as the GBMAs. When the ambiguous situation is removed such that there are no misclassification errors (Figure 5b), controlling for SRE leads to type I error rates that are in accordance with the nominal level (Figure 6b). This result seems to suggest that for multi-ethnic samples, misclassification errors have a more significant effect on the performance of SRE as a control variable than the actual ancestry proportion values themselves. We also noted that the type I error rate associated with the (2, 1) effect size is about 3 times higher than the rate observed with (1, 2). As explained in the methods section, these vectors represent the effect sizes associated with the first 2 components of the ancestry proportion vector that we used to simulate the trait. Based on the symmetry observed on all 4 valid regions identified in figure 5a, one would expect the same amount of type I error inflation for the 2 sets of effect sizes. However, the distribution of allele frequencies in the 3 ancestral populations appears to also affect the degree of confounding that occurs, such that the type I error inflation due to misclassification errors is worst for some cases than it is for others. For example, when we changed the allele frequency of marker G_2 _from (0.2, 0.4, 0.6) to (0.4, 0.2, 0.6) to make it independent but identically distributed with G1 then the observed type I error rate dropped from 0.165 to 0.07. We repeated the same experiment by keeping the frequency of G1 fixed and changing the frequency of G2 to (0.1, 0.3, 0.5). The observed type I error was 0.18; as demonstrated above, by changing the order of the first 2 components of the allele frequency vector, this type I error is reduced to 0.065. The type I error associated with the scenario described in figure 5b remained around the preset threshold of 0.05 independently of the choice of allele frequencies in the 3 ancestral populations.

**Figure 7:**
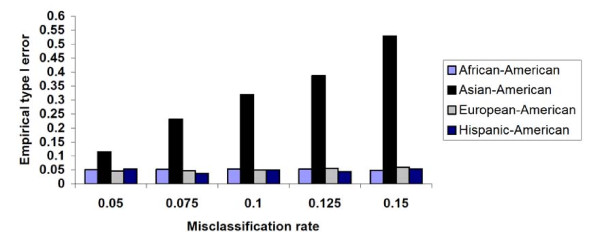
**Effect of misclassification error in SRE in a multi-ethnic sample**. Not all misclassification errors have the same cost. The SRE Chinese American cluster is so well defined in the MESA data that misclassification errors involving them appear to be more costly in terms of the type I error rate.

We also considered the effect of misclassification error between self-reported ethnicity and true ethnicity when the study sample is made of 4 ancestral groups according to their representation in the MESA sample. We looked at the effect of discrepancy between true and self-reported in each ethnic group separately. We observed a small, almost negligible, type I error inflation when a misclassification rate varying between 5% and 15% occurred in exactly one ethnic group for individuals whose self-reported ethnicity is simulated to be European-Americans, African-Americans or Hispanic-Americans. However, significantly higher type I error inflation follows when there are discrepancies between SRE and true ancestry for individuals whose initial ancestry was Asian. This result makes sense intuitively because, as can be seen in Figure 1, the Chinese-American cluster is completely separated from the remaining 3 clusters. A misclassification error involving these individuals is more costly than a misclassification involving the other 3 ethnic groups who all share, albeit at different levels, the European ancestry component. Figure 7 shows the type I error inflation associated with various misclassification rates for each simulated ethnic group. When misclassification occurs for all groups, the type I error inflation can be as high as 11 times the expected level. This type I inflation is again an increasing function of the misclassification rate and seems to depend on the admixture history of the individuals represented in the sample.

## Discussion

We focused on the utility of self-reported ethnicity as a control variable in genetic and epidemiologic studies. We used data collected in the MESA for LVH traits, specifically, LV mass and ejection fraction, to illustrate our points. LVH is one of the strongest determinants of cardiovascular outcomes. Ethnic differences in the distribution of both LV mass and the LV ejection fraction have been reported in many studies, and we found significant evidence of an ethnicity related effect on these phenotypes in the MESA sample.

We observed a high degree of agreement between self-reported ethnicity and two GBMAs computed using genotyped ancestry informative markers. The self-reported Hispanic-Americans were by far the most heterogeneous group represented in this dataset. This result is not surprising given the current definition of the term "Hispanic" which refers to a group of individuals who are culturally and genetically quite diverse. It is now well accepted that the ancestry distribution of self-reported Hispanics reflects, at different degrees, the genetic contribution of the three ancestral populations Africans, Europeans and Native American [27].

Another factor that may explain the genetic heterogeneity detected among the self-reported Hispanics may be the lack of individuals from the Native American ancestral groups represented in the sample. The initial panel of ancestry informative markers used in the MESA study was chosen based on their capacity to distinguish between individuals of Chinese, African and European ancestry. This panel might not be adequate to detect subtle variation between individuals who self identified as Hispanics. Following this analysis, a new panel of markers known to be particularly informative for Hispanics was typed in an effort to better understand the observed variation in the estimation of ancestry in this ethnic group. However, judging by the observed type I error rates, it appears that ancestry proportions estimated with the current marker panel work well as control variables in all the association tests that we have considered.

We could not directly evaluate the type I error on the original sample since it is not known which markers are really under the null hypothesis in that sample. Nevertheless, self reported ethnicity appeared to be effective as a control variable to protect against population stratification and admixture as the genetic background scores and the estimated individual ancestry proportions since we observed significant agreement between the set of markers that show significant p-values independently of the control variable selected for the analysis. The plasmode analysis showed similar results. The type I error was kept at its appropriate level independent of the choice of controls variables, and did show significant inflation when none of them is included in the model. We should note that we did observe a stronger correlation between the p-values obtained when the control variable was estimated using the AIMS than the p-values observed between either of the genetic based measures and SRE. The simulation study shows that, when the number of ancestral populations is equal to 2, even controlling for an individual's true ethnicity might lead to significant type I error inflation depending on the composition of the study sample. We saw that as the gap value was increasing, the performance of true ethnicity was improving and even got very close to the nominal level when the gap was equal to 0.5. However, controlling for the genetic based measure of ancestry led to the correct type I error rates independently of the value of the gap. It is rarely the case that a study participant will report their ethnicity without errors. Self reported ethnicity errors may occur for various reasons, some people may not be fully aware of their true ethnicity while others may identify with one ethnic group despite their admixed background. Therefore, the use of SRE as a control variable when *K = 2 *is discouraged. Our analyses simply suggest that there may be cases where SRE might provide adequate type I error control in the presence of population stratification. This MESA sample, given its composition, seems to be one of these cases. SRE would not necessarily perform as well in other cases. Investigators, in general, will be best served by allocating part of the resources available for their study to genotype the appropriate set of ancestry informative markers. However, SRE seems to be a valuable alternative in multi-ethnic samples when the misclassification rate is likely to be small. This observation is particularly true for smaller studies like candidate gene and other replication studies where a relatively small number of markers are being considered. It is also not unexpected; for example, Walcholder et al. [28] used the law of total probability and stratification to show that the bias due to the confounding effect of admixture decreased with the number of ancestral populations that intermated to lead to the admixed population under consideration. They showed using simulated data that the relative bias when *K *= 3 was between 0.95 and 1.05. We have treated SRE and genetic ancestry as confounders in association tests. We should note that they could also act as effect modifiers depending of the context. However, this distinction cannot be done by a simple test statistic. More information about the causal pathway is needed.

## Methods

The MESA study was designed to investigate the determinants and progression of subclinical cardiovascular diseases in 4 ethnic groups enrolled from six geographic regions in the United States [29]. Institutional Review Boards at each of the six MESA centers where participants were seen for clinical exams reviewed and approved the conduct of the MESA study including genetics research. The sample of 6,814 individuals was drawn such that it contains roughly equal proportions of men and women, all of whom were free of clinically recognized cardiovascular disease at enrollment. Race was self-reported, about 23% of the subjects enrolled in the study self-identified as AA, 11% as CA, 38% as EA and 28% as HA. An intensive evaluation was conducted at baseline, where information regarding height, weight, waist circumference, smoking history, alcohol intake, education level, physical activity, medication, hypertension, heart rate, diabetes, and cholesterol level were collected, among other variables. Data on LV mass and LV ejection fraction were obtained via magnetic resonance imaging (MRI) from MESA participants who consented to the cardiac MRI scan. The LV ejection fraction was calculated by dividing the individual stroke volume by the end-diastolic volume. LV mass was adjusted for body surface area calculated using the formula provided by Wang et al. [21,30]. As will be seen in the results section, these two measures are differently distributed in the four ethnic groups. We will use these ethnic differences in our simulations to create confounding situations that we will seek to control for with three measures of individual ancestry that we describe below.

As part of a MESA genotyping project, ninety-six AIMs were initially genotyped on more than 2,848 participants. These AIMs were selected from an Illumina proprietary SNP database, and were selected to maximize the difference in allele frequencies between the following pairs of ancestral populations: African vs. Chinese, African vs. European and Chinese vs. European.

Utilizing a plasmode generation approach, we developed a resampling procedure, which generates datasets where the null hypothesis of no association holds between each marker and the phenotypes of interest. A plasmode is similar to a simulated dataset; however, a plasmode dataset uses genotypic and phenotypic information observed in a study to construct pseudo-phenotypes so that the 'truth' of the data generating process is known [13,14]. In this case, the relationship among the covariates remained intact while adding the permutated residual to the outcome variable ensured that all tests are conducted under the null hypothesis of no association between the genetic marker and the phenotype of interest. Therefore, we are sure that any observed significant association constitutes a type I error. Finally, we ran a series of simulation studies whose objectives are to better understand our findings and to determine whether they could be further generalized. These simulations are described in the simulation section.

In the remainder of the next section, we discuss the clustering method that was used to group MESA participants based on their observed genotypes, present the statistical approach retained for testing for association between each AIM and the 2 phenotypes of interest, and described the simulation procedures in detail.

### Classification scheme

We used the 'cluster' and 'tree' procedures in the SAS software (version 9.1) to create 4 clusters based on the principal components computed from the 96 AIMs. We applied Ward's minimum variance method and the K-means clustering algorithms to identified clusters of individuals who similar ancestry proportions. To assess the agreement between self-reported ancestry and these clusters, we used Cohen's weighted kappa [31]. Bhapkar's test [32] was applied to test for marginal homogeneity, and log-linear models to test for quasi-symmetry of the data shown in Table 1. This is done to attest the degree, direction and cause (chance or real) of agreement between SRE and the 4 clustered created from the GBS.

### Genetic association tests

We examined all the available AIMs for association with both body surface-adjusted (BSA) LV mass, and LV ejection fraction using 3 different control variables: SRE, ancestry proportion estimates computed using STRUCTURE and genetic background measures computed using principal component analysis. That is, we implemented multivariate linear regression, including (1) SRE as a categorical variable, (2) the proportion of African, Chinese and European ancestry estimated using STRUCTURE, or the first 4 principal components computed using the AIMs data as covariates. We also included gender, income, education level, smoking history, alcohol use, systolic blood pressure, diastolic blood pressure, body mass index, and waist circumference as covariates in each model. Both analyses are conducted using generalized linear models. They also both fall under the structured association test (SAT) framework, which consists in testing for genetic association controlling for a genetic based measure of ancestral background [33]. The SAT approach is commonly used to correct for population stratification and admixture in genetic association tests. Recently published papers have shown that SAT approaches may fail to provide nominal type I errors for various reasons, including measurement error in the estimation of the confounding effect [34,35] and cases where the estimated confounder captures an insufficient fraction of phenotypic variation [36]. However, we restricted our attention to older and better-known SAT approaches [33,37-40]. As a preliminary analysis, we tested the null hypothesis of no association between the selected AIM and the phenotype of interest adjusting for the control variables mentioned above. Unlike in simulation-based work (see next section) the true association of the AIMs with LV mass is not known, and type I error rates for these tests could not be directly evaluated. However, marked differences between the statistical significance observed with each control variable may provide valuable insight regarding their performance. We devised a resampling procedure that guarantees that the methods are being compared under the null hypothesis. Details about this simulation procedure can be found in the next section. Finally, we ran two completely *in silico *analyses, which are also described in the next section, to mimic candidate gene association tests controlling for both SRE and a genetic based measure of ancestral background in order to gain a better understanding of the results observed in the initial test and the plasmode analysis.

### Simulation studies

The first set of simulations is a resampling procedure that guarantees that the control variables are being compared under the null hypothesis of no association between each marker and the phenotype of interest. The second set of simulations seeks to expand upon the results observed under the first set of simulations by identifying the conditions under which accounting for ethnicity (either true or self-reported) is likely to provide appropriate type I error control and evaluating the effect of misclassification errors of SRE in genetic association studies.

#### Resampling procedure

Let *N *denote the number of subjects in the dataset. Let *α *denote the nominal type 1 error rate. Let *Y *denote the phenotype. Let *K *denote the number of AIMs. Let *X *denote the genotype at the marker being considered. Our resampling procedure is as follows:

#### Simulation 1

For *iter = 1 to iterations *{

For *i = 1 to K *{(for each marker)

Regress *Y *on *X *plus all relevant covariates except the confounding variables. When the looping variable *i *takes the value *s (1*≤=*s*≤*K)*, *X *will correspond to the *s^th ^*marker. This regression is fitted using the generalized linear model.

For *j *= 1 to *N *{(for each observation)

compute ${\hat{e}}_{j}=Y_{j}-{\hat{Y}}_{j}$, where ${\hat{Y}}_{j}$ is the predicted value for the *j^th ^*person.

}

Sort the ${\hat{e}}_{j}$'s

Compute a new pseudo-phenotype ${\tilde{Y}}_{j}={\hat{Y}}_{j}+{\hat{e}}_{\left[j\right]}$, where [*j*] denotes the new order after sorting the residuals.

For *m = 1 to K *{(for each marker)

If (*m *≠ *i*) {

Regress $\tilde{Y}$ on X plus the same relevant covariates and each confounding variable.

Compute the Wald test p-value for the regression coefficient of *X*. Denote the resulting p-value as *p_m_*.

}

}

Compute $T_{i}^{iter}\equiv \frac{1}{K-1}\sum_{m\neq i}^{K}I(p_{m}<\alpha )$.

}

Compute $T_{iter}\equiv \frac{1}{K}\sum_{i=1}^{K}T_{i}^{iter}$

*T_iter _*is then an estimate of the type I error rate for the current iteration. Results for 10,000 iterations are summarized in Table 3 for LV mass and Table 4 for LV ejection fraction.

### Description of the second set of simulation procedures

As can be seen in Tables 3 and 4, SRE appears to perform as well as the other GBMAs. The second set of simulations is designed to further elucidate why SRE, despite its much-publicized shortcomings of not being able to adequately control for confounding due to population stratification and admixture, seems to provide the correct type I error rate in this dataset.

First, we considered the case where the confounder is univariate. This could arise when the study sample comprised admixed individuals born from intermating between exactly two ancestral populations. We evaluated how the performance of SRE as a control variable depends on the distribution of ancestry proportions in the sample. Specifically, we wanted to see how the continuity and the size of the gap in a discontinuous ancestry proportion distribution would affect the performance of SRE. Note that a continuous distribution would have a gap of zero. The gap for a discontinuous distribution is defined as the range of the discontinuity region. For example, admixture is an ongoing process. A sample of admixed individuals can comprise individuals who are at different stage of the admixture process. Therefore, it is possible to recruit a sample that can be divided into 2 subsets of individuals: one with very high level of European ancestry and the other with very low level European ancestry. If the minimum European ancestry in the first subset is 0.8 and the maximum European ancestry in the second subset is 0.2 then gap value would simply be (0.8-0.2) = 0.6. We also looked at the effect of various misclassification rates in these association tests.

#### Simulation 2 (univariate ancestry proportion distribution (K = 2))

Let *N *= 2000 be the total number of individuals

For gap = 0.05 to 0.5 by 0.05{

Draw individual ancestry proportion *x *from $\frac{1}{2}U\left(0,f\right)+\frac{1}{2}U\left(1-f,1\right)$, where $f=\frac{1}{2}\left(1-gap\right)$

Set true ethnicity to 1 if *x *∈[0,*f*] and 2 otherwise.

Let *m *represent the misclassification rate.

For *m *= 0.025 to 0.15 by 0.025{

Draw the random variable *s *from a Bernoulli (*m*).

If (*s *= 0) then SRE = true ethnicity

else change the true ethnicity so that a misclassification occurs.

}

Compute $p_{s}^{adx}=xp_{s1}+\left(1_{N}-x\right)p_{s2}$ where *p*_*s***1 **_and *p*_*s***2 **_represent the allele frequency of the *s^th ^*marker in each ancestral population respectively, **1**_*N *_is vector of ones, and $p_{s}^{adx}$ denotes the vector of allele frequencies in the admixed population for the *s^th ^*marker. We consider 2 markers *G_1 _*and *G_2_*. Draw *G*_**1 **_from *Binomial *(2,$p_{1}^{adx}$) and *G*_**2 **_from *Binomial *(2, $p_{2}^{adx}$). That is, we use the simulated ancestry proportion and the allele frequencies in the 2 ancestral populations to generate the genotypic probability for each individual under Hardy Weinberg equilibrium. Note that *G*_**1 **_and *G*_**2 **_are independent conditional on the individual ancestry. We will use *G*_**1 **_to generate the trait and *G*_**2 **_to test for genetic association.

Compute *y *= *α_0 _+ α*_1_*G*_1 _*+ e *where *e ~ N *(**0**, *σ*^2^).

## Note that we set *α*_**1 **_and *σ *such that the effect size is equal to 0.5 in Figure 3a and 1 in Figure 3b.

## Note that conditional on the individual ancestry, the random variable *Y *is also independent of *G_2_*.

Fit the following 3 linear regressions:

1. *y *= *β*_0 _+ *β*_1_*x + β*_1_*G*_2 _+ *e*

2. *y *= *β*_0 _+ *β*_1_*ethn + β*_2_*G*_2 _+ *e*

3. *y *= *β*_0 _+ *β*_1_*sre + β*_2_*G*_2 _+ *e*

Test whether *β*_2 _is statistically significant than 0 at the 0.05 level in each case.

## A statistically significant association observed between *Y *and *G_2 _*will constitute a type I error.

}

Repeat the experiment 10,000 times for each configuration of the gap value and the misclassification rate and count the proportion of times that the parameter *β*_2 _is statistically significant for each control variable. These proportions for the ancestry proportion and SRE regressions are shown in Figure 3. However, we do show the effect of various misclassification rates for 3 gap values (0.05, 0.3 and 0.5) in Table 5.

#### Simulation 3 (multivariate ancestry proportion distribution (K > 2))

We wanted to determine how SRE, when used as a control variable against population stratification, would perform in a multivariate setting. That is, when the number of number of ancestral populations is greater than 2. This simulation procedure resembles the previous one, except that the individual ancestry proportions are drawn from a Dirichlet distribution. We used a different set of parameters for each ethnic group in order to create the conditions needed for confounding to occur. The parameter used to generate the Dirichlet distribution can be represented by a 4 × 4 matrix, where the rows represent the expected individual ancestry proportions in each ethnic group, and for a fixed row, the columns represents the expected individual ancestry proportion from each ancestral population.

1) Let *N *= 2000 individuals divided equally into 4 subsets such that *n_k _*= 500 for *k *= 1,2,3,4 is the sample size in each ethnic group.

2) Let the SRE for an individual in the *k^th ^*subgroup be *k*.

3) We considered 4 cases; the parameters used for the Dirichlet distribution in each case are chosen as follows:

(a) Proportions that are very close to ancestry proportion estimates obtained with STRUCTURE in the MESA sample;

(b) Values near a 4 × 4 identity matrix with diagonal elements equal to 0.97 and off diagonal elements equal to 0.01;

(c) Values very close to what would be observed in equally admixed individuals, that is all proportions are set 0.25;

(d) Different ancestry proportions where the contribution of one specific ancestral population is clearly greater in each admixed population. That is the diagonal elements are set to 0.55 and off diagonal elements at 0.15 and made sure that the row and column sums are equal to 1.

4) Let *P*_**1 **_= (0.1, 0.5, 0.3, 0.9) and *P*_**2 **_= (0.05, 0.25, 0.50, 0.75) be the frequency of the reference allele of the markers *G*_**1 **_and *G*_**2 **_respectively in each ancestral population. These frequencies were chosen arbitrarily with the only constraint being that they vary greatly between the 4 ancestral populations. Confounding will occur if the distribution of the trait is also different in the 4 ancestral populations.

5) The allele frequencies of *G*_**1 **_and *G*_**2 **_in the admixed population ($p_{1}^{adx}$,$p_{2}^{adx}$) are computed as the weighted averages of the allele frequencies given in (4), where the weights are the simulated ancestry proportions drawn for the Dirichlet distribution.

6) Draw *G*_**1 **_and *G*_**2 **_according to these averages.

7) The outcome variable *Y *is simulated from a normal distribution where the parameters are based on the distribution of the LV ejection fraction observed each ethnic group observed in the MESA study. That is, we used the mean in each group as the intercept in the model described in the next step. We then compute the pooled variance and set $\beta_{1}=\beta_{2}=\beta_{3}=\frac{\sigma }{2}$ such that each component has an effect size of 0.5, where *σ*^2 ^represents the pooled variance.

8) The remaining steps are similar to those taken in simulation 2. That is, we fitted the following linear regressions:

1. *y *= *β*_0 _+ *β*_1_*x*_1 _+ *β*_2_*x*_2 _+ *β*_3_*x*_3 _+ *β*_4_*G*_2 _+ *e*. The variables *x_1_*, *x_2 _*and *x_3 _*are the first 3 components of the individual ancestry proportion vector drawn from the Dirichlet distribution.

2. *y *= *α*_0 _+ *α*_1_*SRE *+ *α*_2_*G*_2 _+ *e*. The variable *SRE *has 4 levels, which are defined in step 2. Therefore, *α_1 _*is a vector with 4 components.

We then tested whether *β*_4 _and *α*_2 _are statistically different than 0 at the 0.05 level in each model, and repeated each experiment 10,000 times. The results of this simulation procedure can be seen in Figure 4. As can be seen in this figure, when the sample contained admixed individuals with more than 2 ancestral populations, SRE performed rather well as a control variable. This suggests that it is not the variations in the ancestry proportions themselves that cause the type I error inflation.

To better understand when the use of SRE as a control variable may fail, we devised a situation where it may be unclear which ethnicity to assign to individuals whose ancestry proportions take specific values. To facilitate the graphical representation of each scenario, we focused on the case where there are exactly 3 ancestral populations. In this case, an individual's ancestry proportion can be represented by a vector with 3 components, *adx*_1_, *adx*_2 _*and adx*_3 _such that $\sum_{i=1}^{3}adx_{i}=1$. Therefore, without loss of generality, we can restrict our attention to a bivariate distribution by focusing only on the first two components of this vector. As can be seen in Figure 5, the choice of gap values on both the x-axis and y-axis and in addition to the constraint that *adx*_**1 **_+ *adx*_**2 **_≤ **1**, define 3 or 4 specific regions. Figure 5a shows 4 valid regions, and if one decides to assign ethnicity according to the maximum value of the vector (*adx*_**1**_, *adx*_**2**_, *adx*_**3**_), it is not exactly clear what the correct ethnicity assignment should be for the individuals whose ancestry proportions fall in region IV. There is no such ambiguity in Figure 5b.

The simulation steps are similar to those described in simulation 2, except that there are now two gap values: one for *adx*_**1 **_and one for *adx*_**2**_. The ancestry proportions are each drawn independently from a uniform distribution, which has been rescaled such that the proportions add up to 1. We then excluded the ancestry proportions that fell in the regions defined by the gaps. In Figure 5a, we excluded values of *adx*_**1 **_and *adx*_**2 **_that fall between 0.1 and 0.3. The range of excluded values went from 0.1 to 0.5 in 5b. As in all previous cases, we considered 2 markers G1 and G2. We used G1 to simulate the trait, and G2 to test for association with the simulated trait. All significant association is seen as type I error. We use the vector (0.4, 0.2, 0.6) as the allele frequency in the 3 ancestral populations for G1 and (0.2, 0.4, 0.6) for G2. We also considered various effect sizes for evaluating the contribution of admixture in the confounding pathway. We also changed the allele frequencies vector to account for the fact that *k*, the number of ancestral populations, is now 3 instead of 4. The remaining simulation steps are again similar to those described in simulation 3. The results of this simulation analysis can be seen on Figure 6, where the vector (*a*,*b*) represents the coefficient associated with the variables *adx*_**1 **_and *adx*_**2 **_in the model. The error term in all models is drawn from a normal distribution with 0 and variance 1 such that the effect size associated with *adx*_**1 **_and *adx*_**2 **_are equal to *a *and *b *respectively.

#### Simulation 4 (effect of misclassification error on SRE when K = 4)

As can be seen in Figure 7 when the number of ancestral population is greater than 2, it is misclassification errors, as opposed to the actual distribution of the ancestry proportions that dictate the performance of SRE as a control variable. We ran a final simulation study to evaluate the effect of misclassification errors on a multi-ethnic sample like the MESA. The simulations steps are very similar to those taken in simulation 3.

1) Let *n_k _*= 500 for *k *= 1,2,3,4 be the sample size in each ethnic group.

2) Let the true ethnicity of any individual in the *k^th ^*subgroup be *k*.

3) Draw *x_k _*from *Dir*(*α_k_*) where *α_k _*is based on the ancestry proportions observed in the MESA study. These proportions are displayed in Figure 2.

That is, (**0.09, 0.02, 0.84, 0.05**) for the African-Americans, (**0.89, 0.02, 0.02, 0.07**) for the European-Americans, (**0.24, 0.05, 0.14, 0.57**) for the Hispanic-Americans and (**0.01, 0.97, 0.01, 0.01**) for the Chinese-Americans.

To evaluate the effect of misclassification errors on the performance of SRE as a control variable, the true ethnicity of a fraction *m *of individuals in each subgroup is changed to create misclassification in the SRE variable. This fraction is assigned to one of the 3 remaining subgroups uniformly. This is done for each subgroup separately.

4) We let *m *vary from 0.05 to 0.15 by 0.025.

5) The remaining simulations steps are similar to those taken in simulation 3, and are not repeated here.

## List of abbreviations

MESA: Multi-Ethnic Study of Atherosclerosis; SRE: self-reported ethnicity; GBMA: genetic based measures of ancestry; AIMs: ancestry informative markers; IAP: individual ancestry proportion; GBS: genetic background score; AA: African-Americans; CA: Chinese-Americans; EA: European-Americans; HA: Hispanic-Americans; LVH: Left ventricular hypertrophy; LV: Left ventricular; MRI: magnetic resonance imaging; BSA: body surface-adjusted; SAT structured association test.

## Authors' contributions

JD conceived the manuscript, conducted the analyses, interpreted the results and drafted the manuscript. DTR and DBA helped conceive the simulation experiments, interpret the results and edit the manuscript. KMR, LKV and MAP helped during the analyses and reviewed the manuscript. DAB, JHY and DKA made the data available, helped interpret the results and reviewed the manuscript. All authors have read and approved the manuscript.

## References

[B1] Burchard EG Ziv E Coyle N Gomez SL Tang H Karter AJ The Importance of Race and Ethnic Background in Biomedical Research and Clinical Practice N Engl J Med 2003 348 1170 1175 10.1056/NEJMsb025007 12646676

[B2] Witzig R The Medicalization of Race: Scientific Legitimization of a Flawed Social Construct Ann Intern Med 1996 125 675 679 8849153 10.7326/0003-4819-125-8-199610150-00008

[B3] Cooper RS Kaufman JS Ward R Race and genomics N Engl J Med 2003 348 1166 1170 10.1056/NEJMsb022863 12646675

[B4] Sinha M Larkin EK Elston RC Redline S Self-Reported Race and Genetic Admixture N Engl J Med 2006 354 421 422 10.1056/NEJMc052515 16436780

[B5] Burnett MS Strain KJ Lesnick TG de Andrade M Rocca WA Maraganore DM Reliability of Self-reported Ancestry among Siblings: Implications for Genetic Association Studies Am J Epidemiol 2006 163 486 492 10.1093/aje/kwj057 16421243

[B6] Risch N Dissecting Racial and Ethnic Differences N Engl J Med 2006 354 408 411 10.1056/NEJMe058265 16436773

[B7] Pritchard JK Stephens M Donnelly P Inference of population structure using multilocus genotype data Genetics 2000 155 945 959 10835412 10.1093/genetics/155.2.945 PMC1461096

[B8] Tang H Quertermous T Rodriguez B Kardia SLR Zhu XF Brown A Genetic structure, self-identified race/ethnicity, and confounding in case-control association studies Am J Hum Genet 2005 76 268 275 10.1086/427888 15625622 PMC1196372

[B9] Wilson JF Weale ME Smith AC Gratrix F Fletcher B Thomas MG Population genetic structure of variable drug response Nat Genet 2001 29 265 269 10.1038/ng761 11685208

[B10] Liu XQ Paterson AD John EM Knight JA The role of self-defined race/ethnicity in population structure control Ann Hum Genet 2006 70 496 505 10.1111/j.1469-1809.2005.00255.x 16759181

[B11] Rosenberg NA Pritchard JK Weber JL Cann HM Kidd KK Zhivotovsky LA Genetic structure of human populations Science 2002 298 2381 2385 10.1126/science.1078311 12493913

[B12] Falush D Stephens M Pritchard JK Inference of population structure using multilocus genotype data: Linked loci and correlated allele frequencies Genetics 2003 164 1567 1587 12930761 10.1093/genetics/164.4.1567 PMC1462648

[B13] Mehta T Tanik M Allison DB Towards sound epistemological foundations of statistical methods for high-dimensional biology Nat Genet 2004 36 943 947 10.1038/ng1422 15340433

[B14] Vaughan LK Divers J Padilla MA Redden DT Tiwari HK Pomp D The use of plasmodes as a supplement to simulations: A simple example evaluating individual admixture estimation methodologies Comput Stat Data Anal 2009 53 1755 1766 10.1016/j.csda.2008.02.032 20161321 PMC2678733

[B15] Bielen E Fagard R Amery A The Inheritance of Left-Ventricular Structure and Function Assessed by Imaging and Doppler Echocardiography Am Heart J 1991 121 1743 1749 10.1016/0002-8703(91)90021-9 2035387

[B16] Koren MJ Mensah GA Blake J Laragh JH Devereux RB Comparison of left-ventricular mass and geometry in black and white patients with essential hypertension Am Heart J 1993 6 815 823 10.1093/ajh/6.10.815 8267936

[B17] Ghali JK Liao Y Cooper RS Left ventricular hypertrophy in the elderly Am J Geriatr Cardiol 1997 6 38 49 11416436

[B18] Okin PM Devereux RB Jern S Kjeldsen SE Julius S Nieminen MS Regression of electrocardiographic left ventricular hypertrophy during antihypertensive treatment and the prediction of major cardiovascular events JAMA 2004 292 2343 2349 10.1001/jama.292.19.2343 15547161

[B19] Kong CH Farrington K Determinants of left ventricular hypertrophy and its progression in high-flux haemodialysis Blood Purif 2003 21 163 169 10.1159/000069155 12601259

[B20] Tang W Arnett DK Province MA Lewis CE North K Carr JJ Racial Differences in the Association of Coronary Calcified Plaque with Left Ventricular Hypertrophy: The National Heart, Lung, and Blood Institute Family Heart Study and Hypertension Genetic Epidemiology Network Am J Cardiol 2006 97 1441 1448 10.1016/j.amjcard.2005.11.076 16679080

[B21] Natori S Lai S Finn JP Gomes AS Hundley WG Jerosch-Herold M Cardiovascular Function in Multi-Ethnic Study of Atherosclerosis: Normal Values by Age, Sex, and Ethnicity Am J Roentgenol 2006 186 S357 S365 10.2214/AJR.04.1868 16714609

[B22] Drazner MH Dries DL Peshock RM Cooper RS Klassen C Kazi Farhana Left ventricular hypertrophy is more prevalent in blacks than whites in the general population: The Dallas Heart Study Hypertension 2005 46 124 129 10.1161/01.HYP.0000169972.96201.8e 15939807

[B23] Gardin JM Wagenknecht LE Antonculver H Flack J Gidding S Kurosaki T Relationship of Cardiovascular Risk-Factors to Echocardiographic Left-Ventricular Mass in Healthy-Young Black-And-White Adult Men and Women - the Cardia Study Circulation 1995 92 380 387 7634452 10.1161/01.cir.92.3.380

[B24] Heckbert SR Post W Pearson GDN Arnett DK Gomes AS Jerosch-Herold M Traditional Cardiovascular Risk Factors in Relation to Left Ventricular Mass, Volume, and Systolic Function by Cardiac Magnetic Resonance Imaging: The Multiethnic Study of Atherosclerosis Am J Cardiol 2006 48 2285 2292 10.1016/j.jacc.2006.03.072 PMC1794681 17161261

[B25] Parra EJ Kittles RA Shriver MD Implications of correlations between skin color and genetic ancestry for biomedical research Nat Genet 2004 36 S54 S60 10.1038/ng1440 15508005

[B26] Knowler WC Williams RC Petitt DJ Steinberg AG Gm3-5,13,14 and Type-2 Diabetes-Mellitus - An association in American-Indians with genetic admixture Am J Hum Genet 1988 43 520 526 3177389 PMC1715499

[B27] Bertoni B Budowle B Sans M Barton SA Chakraborty R Admixture in Hispanics: Distribution of ancestral population contributions in the continental United States Hum Biol 2003 75 1 11 10.1353/hub.2003.0016 12713142

[B28] Wacholder S Rothman N Caporaso N Population stratification in epidemiologic studies of common genetic variants and cancer: quantification of bias J Natl Cancer Inst 2000 92 1151 1158 10.1093/jnci/92.14.1151 10904088

[B29] Bild DE Bluemke DA Burke GL Detrano R Diez Roux AV Folsom AR Multi-Ethnic Study of Atherosclerosis: Objectives and Design Am J Epidemiol 2002 156 871 881 10.1093/aje/kwf113 12397006

[B30] Wang Y Moss J Thisted R Predictors of body surface area J Clin Anesth 1992 4 4 10 10.1016/0952-8180(92)90111-D 1540367

[B31] Cohen J A coefficient of agreement for nominal scales Educational and Psychological Measurement 1960 20 37 46 10.1177/001316446002000104

[B32] Bhapkar VP A note on the equivalence of two test criteria for hypotheses in categorical data JASA 1966 61 228 235

[B33] Redden D Divers J Vaughan L Tiwari H Beasley T Fernandez J Regional admixture mapping and structured association testing: conceptual unification and an extensible general linear model Plos Genetics 2006 2 1254 1264 10.1371/journal.pgen.0020137 PMC1557785 16934005

[B34] Divers J Vaughan LK Padilla MA Fernandez JR Allison DB Redden DT Correcting for measurement error in individual ancestry estimates in structured association tests Genetics 2007 176 1823 1833 10.1534/genetics.107.075408 17507670 PMC1931538

[B35] Kimmel G Jordan MI Halperin E Shamir R Karp RM A Randomization Test for Controlling Population Stratification in Whole-Genome Association Studies Am J Hum Genet 2007 81 895 905 10.1086/521372 17924333 PMC2265648

[B36] Epstein MP Allen AS Satten GA A Simple and Improved Correction for Population Stratification in Case-Control Studies Am J Hum Genet 2007 80 921 930 10.1086/516842 17436246 PMC1852732

[B37] Zhang SL Zhu XF Zhao HY On a semiparametric test to detect associations between quantitative traits and candidate genes using unrelated individuals Genet Epidemiol 2003 24 44 56 10.1002/gepi.10196 12508255

[B38] Price AL Patterson NJ Plenge RM Weinblatt ME Shadick NA Reich D Principal components analysis corrects for stratification in genome-wide association studies Nat Genet 2006 38 904 909 10.1038/ng1847 16862161

[B39] Pritchard JK Stephens M Rosenberg NA Donnelly P Association mapping in structured populations Am J Hum Genet 2000 67 170 181 10.1086/302959 10827107 PMC1287075

[B40] Pritchard JK Stephens M Donnelly PJ Correcting for population stratification in linkage disequilibrium mapping studies Am J Hum Genet 1999 65 A101 10.1086/302449

